# The Special Issue “The 20th Anniversary of Pharmaceuticals—Multi-Targeted Natural Products as Therapeutics” Editorial—Multi-Targeted Therapeutics from Natural Sources: What Do We Know?

**DOI:** 10.3390/ph18040442

**Published:** 2025-03-21

**Authors:** Elena Y. Enioutina, Katheleen M. Job, Catherine M. Sherwin

**Affiliations:** 1 The Division of Clinical Pharmacology, Pediatrics, The Spencer Fox Eccles School of Medicine, Salt Lake City, UT 84108, USA; kate.job@hsc.utah.edu; 2 Internal Medicine, UWA Medical School, The University of Western Australia, Perth, WA 6009, Australia; catherine.sherwin85@gmail.com

Herbal and marine products in the form of extracts, infusions, and decoctions have been used for centuries in folk and traditional medicine. Preparations typically contain multiple biologically active compounds. The World Health Organization (WHO) estimated that about 65% of consumers worldwide use natural remedies containing bioactive molecules to treat various diseases [1]. Most single-molecule drugs of natural origin were developed in the middle of the 20th century through the active screening of natural products for bioactive molecules [2]. Examples include artemisinin, cyclosporine, taxol, doxorubicin, and many others. However, at the beginning of the 21st century, we saw a decline in the number of “natural drugs” introduced to the market. A limiting factor could be explained by the pharmaceutical industry blockbuster model, implying the development of few drugs that make a bulk profit (e.g., USD 1 billion in sales for a drug per year [2]). Another reason for this decline could be the introduction of the high-throughput screening of synthetic libraries. A limitation to the large-scale production of “natural drugs” is the availability of herbal or marine sources for their production. However, the research community has expressed great interest in investigating new therapeutics from natural sources. The Special Issue “The 20th Anniversary of Pharmaceuticals—Multi-Targeted Natural Products as Therapeutics” has had great success, with 25 articles published. These comprise eight review articles and seventeen research articles. This Special Issue aimed to summarize and examine the latest research findings in identifying natural products targeting multiple pathways and body functions that can successfully treat multifactorial diseases. The Guest Editors are thankful for all contributors.

The results of the analysis of studies published in this Special Issue showed that many bioactive compounds in natural products affect various body functions or target multiple pathways. A combination of bioactive molecules in an extract or decoctions usually affects various body functions. Interestingly, some molecules belonging to the same chemical class may target different pathways, often resulting in potential therapeutic effects [Contributions 1–3]. Torres-Sanchez A. et al., for example, reported that pentacyclic triterpenes stimulate human lung carcinoma cell apoptosis, S-phase and G2/M-phase cycle arrest, and the downregulation of MAPK/PI3K and STAT3 gene expression [Contribution 1]. Lung cancers are some of the most common malignancies worldwide, with a poor survival rate [3]. Multiple new treatment strategies have been proposed, from nanoparticle drug delivery to malignant cells and minimally invasive photothermal therapy to the molecular targeting of pathways and receptors involved in malignant cell expansion. The addition of pentacyclic triterpenes as an adjuvant therapy to already existing treatment regimens could significantly improve the survival rate and life satisfaction of treated patients.

Some articles in this Special Issue also focused on the effects of bioactive molecules on immune system functions [Contributions 4–8] [4]. Over the past decade, numerous publications have demonstrated the immunomodulatory activities of traditional medicine preparations and their biologically active components [5,6,7,8,9]. For example, the immunomodulatory properties of ginsenosides isolated from *Panax notoginseng* were found to depend on the ginsenosides’ structure and can demonstrate immunosuppressive or immunostimulatory properties [6]. Glycoprotein isolated from *Dioscorea batatas* Decne activated macrophages through the TLR-4 receptor and stimulated nuclear factor-κB (NF-kB), c-Jun N-terminal kinase, and several other pathways, resulting in the production of IL-1β, IL-6, and TNF-α [10]. The bark of *Dioscorea batatas* Decne demonstrated anti-inflammatory activity by inhibiting inducible nitric oxide synthase (iNOS) and Cyclooxygenase-2 (COX-2) expression in LPS-activated RAW264.7 cells via the suppression of NF-κB p65 subunit nuclear translocation [11]. Catechins from green tea modulated the activity of T cells [12]. Gasmi A. et al. reviewed the immunomodulatory properties of polyphenols, terpenoids, β-glucans, and vitamins [Contribution 4, 29 citations as of 30 January 2025]. Beta-glucans activate phagocytic cells (e.g., neutrophils, monocytes, and macrophages), antigen-presenting dendritic cells, Natural Killer (NK) cells through scavenger receptors, Dectin-1, complement receptor 3, and Toll-Like Receptors (TLRs) 2/6 [13,14]. Additionally, β -glucans demonstrate anti-angiogenic properties, which, combined with activated cytotoxic NK cells, may reduce tumor proliferation and metastasis development [14]. Glucans, as natural polysaccharides, could also be valuable as drug-delivery vessels. The data presented in Contribution 6 indicated that cannabidiol extract (CBD) reduces IL-5 and IL-13, which are key cytokines involved in asthma development. An animal model of asthma showed that CBD treatment reduced the levels of IgE and asthma-associated cytokines alongside lung infiltration by immune cells.

Medicinal herbs or their bioactive compounds often simultaneously display immunomodulatory and antimicrobial activity, which can be particularly advantageous in this era of multidrug-resistant bacteria [15]. Herbal extracts often show a broad spectrum of antimicrobial activities against bacteria, viruses, and fungi. Wang et al. investigated the mechanisms behind the antimicrobial activity of walnut green husk extracts against *Staphylococcus aureus*, *Bacillus subtilis*, and *Escherichia coli* [16]. The authors determined that the extracts stimulated intracellular ions and alkaline phosphatase leakage from bacteria, reduced ATPase activity, and upregulated pathways associated with RNA degradation and oxidative phosphorylation. The data presented by Fernandes M. et al. showed that sliver nanoparticles synthesized with the help of extracts from brown algae (*Cystoseira baccata* and *Cystoseira tamariscifolia*) had strong bactericidal or bacteriostatic effects against *Pseudomonas aeruginosa* and *Escherichia coli* [17]. The authors of Contributions 9–12 also focused on the antimicrobial activities of several natural products and their bioactive compounds. The *Kielmeyera membranacea* extract and biflavonoid podocarpus flavone A from *Kielmeyera membranacea* demonstrated anti-*Mycobacterium marinum* activity in vitro and in infected zebrafish [Contribution 12]. Treatment with podocarpus flavone A stimulated the expression of IL-12 and TNF-α cytokines in fish larvae after 5 days of infection. This contribution is especially important due to the growing number of cases of multi-drug-resistant tuberculosis. Hamdi A. et al. screened and ADME-profiled essential oils isolated from *Citrus limon* L. and *Citrus paradisi* L. for their antimicrobial and anti-coagulate activity [Contribution 10]. The essential oils showed bactericidal activity against Gram-positive and Gram-negative bacteria and fungicidal activity against Candida species.

Hepatoprotective and liver function-improving activities are most often associated with silymarin isolated from milk thistle [18,19]. Recently, more studies have investigated the effects of other medicinal herbs on liver functions [20,21,22]. Contributions 13–15 describe the hepatoprotective, anti-lipidemic, anti-glycemic, choleretic, and hepato-regenerative activities of extracts from *Cichorium intybus*, *Hypericum perforatum*, and polyherbal extract from Cassia *auriculata* leaf, *Centella asiatica* leaf, and *Zingiber officinale* rhizome. The data presented in these articles clearly demonstrated that these extracts regulate glucose and lipid metabolism, protect the liver from xenobiotic injury, and may accelerate liver regeneration.

In conclusion, it appears that there is considerable interest in investigating the effects of purified extracts prepared from natural products or bioactive molecules from these extracts. The PubMed search for natural products and therapeutics displayed 33,703 articles, including 7548 reviews published in 2023–2024, suggesting that researchers have expressed considerable interest in investigating new properties of natural products and summarizing knowledge from previous research. The ability of bioactive molecules to affect multiple signaling pathways and improve several body functions has the potential to decrease the use of multiple drugs for the treatment of different conditions. Additionally, such natural drugs can be used as adjuvant therapies along with conventional regimens to provide positive therapeutic outcomes.
